# Multiplatform profiling of pancreatic neuroendocrine tumors: Correlative analyses of clinicopathologic factors and identification of co-occurring pathogenic alterations

**DOI:** 10.18632/oncotarget.27265

**Published:** 2019-10-22

**Authors:** Jun Gong, Edik M. Blais, Joseph R. Bender, Michelle Guan, Veronica Placencio-Hickok, Emanuel F. Petricoin, Michael J. Pishvaian, Gary Gregory, Richard Tuli, Andrew E. Hendifar

**Affiliations:** ^1^Gastrointestinal and Neuroendocrine Malignancies, Samuel Oschin Cancer Center, Cedars-Sinai Medical Center, Los Angeles, CA 90048, USA; ^2^Perthera, Inc, McLean, VA 22102, USA; ^3^George Mason University, Fairfax, VA 22030, USA; ^4^Lombardi Comprehensive Cancer Center, Georgetown University Medical Center, Washington D.C. 20007, USA

**Keywords:** pancreatic neuroendocrine tumors, genomic profiling, proteomic profiling, molecular pathways, co-occurring alterations

## Abstract

**Background:**

Multi-omic profiling of pancreatic neuroendocrine tumors (PanNETs) was performed to correlate genomic, proteomic, and molecular pathway alterations with clinicopathologic factors and identify novel co-occurring pathogenic alterations of potential clinical relevance to PanNET management.

**Methods:**

PanNETs referred to Perthera, Inc. having undergone molecular profiling for precision matched therapeutic purposes were screened. Correlative analyses were performed using Fisher’s exact test across individual pathogenic alterations or altered molecular pathways and clinicopathologic variables. Associations were visualized by hierarchical clustering. Prognostic associations with overall survival (OS) were identified using Cox regression for pathogenic alterations and pathway-level alterations. Hazard ratios (HR) and odds ratios (OR) were reported with 95% confidence intervals (CI).

**Results:**

From 12/2014–1/2019, 46 patients with predominantly locally advanced and metastatic PanNETs were included. *MEN1* alterations by next-generation sequencing (NGS) were less associated with having high-grade PanNETs and metastatic disease at diagnosis (*p* ≤ 0.05). Genomic alterations associated with increased replicative stress (primarily driven by *RB1* and *TP53*) correlated with higher grade (OR 6.87 [95% CI: 1.57-35.18], *p* = 0.0043) and worse OS (HR 13.62 [95% CI: 1.51-122.5], *p* = 0.0198). Other significant associations included: ERCC1 protein expression with *DAXX* or *MEN1* alterations (NGS), *PTEN* (NGS) with *ARID1A* or *TP53* alterations (NGS), and history of diabetes coincided with cell cycle pathway alterations but was mutually exclusive with replicative stress pathway alterations.

**Conclusions:**

We identified several molecular signatures of potential clinical significance for therapeutic targeting and prognostication in PanNETs warranting prospective validation. Our findings are hypothesis generating and can inform larger molecular profiling efforts in PanNETs.

## INTRODUCTION

Pancreatic neuroendocrine tumors (PanNETs) have been established as a molecularly targetable group of cancers with approved inhibitors of mTOR and VEGFR/PDGFR/c-Kit that are available in the treatment paradigm for unresectable or advanced, well-differentiated disease [1, 2]. Comprehensive genomic profiling of 68 non-metastatic and metastatic PanNETs provided amongst the first glimpses of the molecular landscape for this malignancy and identified frequently recurring somatic mutations in *MEN1* (44.1%), *DAXX* (25%), *ATRX* (17.6%)*, TSC2* (8.8%), *PTEN* (7.3%) **, and *PIK3CA* (1.4%) [3].

Recently, the Australian Pancreatic Cancer Genome Initiative (APGI) within the framework of the International Cancer Genome Consortium (ICGC) performed whole-genome sequencing of 102 localized, locally advanced, and metastatic PanNETs to further characterize pathogenic mechanisms and uncover novel mutations for therapeutic targeting [4]. In this seminal study, potentially actionable somatic alterations in 4 core pathways were identified: DNA damage repair (including *MUTYH* (mutational frequency of 6%), *CHEK2* (4%), and *BRCA2* (1%)), chromatin modification (including *MEN1* (41%), *SETD2* (5%), and *MLL3* (5%)), altered telomere length (including *DAXX* (22%) and *ATRX* (10%)), and mTOR signaling (including *PTEN* (7%), *DEPDC5* (2%), *EWSR1* fusion (3%), *TSC1* (2%), and *TSC2* (2%)).

Despite the results of these landmark studies that have provided a framework for defining the most frequently altered genes and molecular pathways in PanNETs, their relationships to clinicopathologic characteristics remain poorly described. In this study, we describe our experience in cataloging alterations identified through multiplatform profiling of a cohort of predominantly locally advanced and metastatic PanNETs. In particular, we focused our analysis on co-occurring alterations of known pathogenicity and correlating molecular and pathway alterations with various clinicopathologic factors. We believe these analyses to be important in further defining molecular signatures and patient subsets of potential clinical relevance to PanNET management.

## RESULTS

### Patient characteristics

From 12/2014–12/2018, a total of 46 patients diagnosed with predominantly locally advanced and metastatic PanNETs were included in this study (Table 1). The median age was 52 years (range 30–79) and the majority of cases (65%) were low or intermediate grade.

**Table 1:** Patient characteristics

**Characteristic (*n* = 46)**	Frequency (%)^*^
Age (median)	52 years (range 30–79)
Gender	
Female	24 (52%)
Male	22 (48%)
Stage^a^	
IV	25 (54%)
III	3 (7%)
II	7 (15%)
I	1 (2%)
Locally advanced	10 (22%)
Grade^b^	
Low/intermediate	30 (65%)
High	16 (35%)
Site of biopsy	
Liver	23 (50%)
Pancreas	18 (39.1%)
Lymph node	2 (4.3%)
Peritoneum	1 (2.2%)
Spleen	1 (2.2%)
Soft tissue	1 (2.2%)

^*^May not total 100% due to rounding.

^a^American Joint Committee on Cancer (AJCC) 7th edition.

^b^WHO criteria.

### Molecular alterations

The most commonly detected somatic alterations including pathogenic variants and VUS by NGS are shown in Table 2. Mutations in *MEN1* (41%), *DAXX* (28%), *TP53* (20%), *PTEN* (18%), *RB1* (13%), and *ARID1A* (11%) comprised the most frequently altered genes in our NGS cohort of 46 patients with PanNETs. PTEN (100%), mismatch repair (MMR) proteins (100%), TOP1 (64%), TUBB3 (64%), pAKT (58%), ERCC1 (27%), and TLE3 (25%) were among those most frequently expressed in our IHC cohort (Table 2). Analysis by *in-situ* hybridization did not identify any significant alterations across genes assayed: *ALK*, *HER2*, *MET*, *ROS1*, and *TOP2A*.

**Table 2:** Frequently detected molecular alterations detected by NGS and IHC

Alteration^*^	**Pathway Association**	**Frequency (%)^+^**
NGS (*n* = 46)		
MEN1	Chromatin remodeling	19 (41%)
DAXX	Chromatin remodeling	13 (28%)
TP53	Replicative stress	9 (20%)
PTEN	mTOR signaling	8 (18%)
RB1	Replicative stress	6 (13%)
ARID1A	mTOR signaling	5 (11%)
TSC2	mTOR signaling	4 (9%)
SETD2	Chromatin remodeling	4 (9%)
ATRX	Replicative stress	3 (7%)
IHC (*n* = 28)		
MMR	Microsatellite status/mismatch repair	25/25 (100%)
PTEN	mTOR signaling	12/12 (100%)
TOP1	Taxane resistance	7/11 (64%)
pAKT	mTOR signaling	7/12 (58%)
TS	5-FU resistance	8/26 (31%)
ERCC1	Platinum resistance	7/26 (27%)
TLE3	Wnt signaling	3/12 (25%)
PD-1	Immune checkpoint	2/10 (20%)
RRM1	Gemcitabine resistance	1/13 (8%)
HER2	HER2 signaling	0/27 (0%)
PD-L1	Immune checkpoint	0/13 (0%)

^*^Includes pathogenic and variants of unknown significance.

^+^Same alteration can occur in more than 1 patient.

NGS, next-generation sequencing; IHC, immunohistochemistry.

### Correlative analyses

Comparisons of molecular alterations and clinicopathologic variables identified several significant correlations (Table 3). Alterations in *MEN1* (next-generation sequencing or NGS) were less associated with having high-grade PanNETs, while alterations in *RB1* (NGS), *TP53* (NGS), and the replicative stress pathway were associated with having high-grade PanNETs. Alterations in *MEN1* (NGS) and age >54 were less associated with metastatic disease at diagnosis. A history of diabetes was significantly associated with having an altered cyclin-dependent kinase (CDK) but less associated with having an altered replicative stress pathway. Several co-occurring alterations reached statistical significance: *RB1* (NGS) and *TP53* (NGS), *MEN1* (NGS) with ERCC1 expression (immunohistochemistry or IHC) *PTEN* (NGS) and *ARID1A* (NGS), *DAXX* (NGS) and ERCC1 (IHC), *PTEN* (NGS) and *TP53* (NGS), and *MEN1* (NGS) with *DAXX* (NGS).

**Table 3:** Significant correlations between molecular alterations and clinicopathologic variables

Variable 1^*^	Variable 2^*^	**OR**^**+**^	**95% CI**	**Fisher’s (two-tailed) *p*-value**
High grade tumor	RB1 (NGS)	Infinite	2.82–Infinite	0.0009
RB1 (NGS)	TP53 (NGS)	12.73	1.43–175.82	0.0095
ERCC1 (IHC)	MEN1 (NGS)	11.68	1.07–639.81	0.0261
High grade tumor	TP53 (NGS)	10.22	1.58–118.17	0.0049
PTEN (NGS)	ARID1A (NGS)	9.92	0.91–147.03	0.0307
History of diabetes	Cyclin-dependent kinase pathway	8.42	0.94–95.00	0.0292
DAXX (NGS)	ERCC1 (IHC)	8.40	0.96–120.72	0.0283
Replicative stress pathway	High grade tumor	6.87	1.57–35.18	0.0043
PTEN (NGS)	TP53 (NGS)	6.23	0.87–47.19	0.0359
MEN1 (NGS)	DAXX (NGS)	5.74	1.23–32.41	0.0171
Metastatic at diagnosis	MEN1 (NGS)	0.22	0.05–0.90	0.0288
Metastatic at diagnosis	Age >54	0.17	0.04–0.72	0.0070
High grade tumor	MEN1 (NGS)	0.13	0.01–0.72	0.0105
History of diabetes	Replicative stress pathway	0	0–0.65	0.0087

^*^Pathogenic alterations only.

^+^For odds ratio >1, variable 1 is more prevalent when variable 2 is present; for odds ratio <1, variable 1 is less prevalent when variable 2 is present.

OR, odds ratio; CI, confidence interval; NGS, next-generation sequencing; IHC, immunohistochemistry; replicative stress pathway (p53, RB1, ATRX, ATM, CCNE1, CHEK2, MYC), chromatin remodeling pathway (ARID1A, ARID2, ASXL1, CHD2, CTCF, DAXX, KMT2D, MEN1, MLL3, PBRM1, SETD2, TET2), cyclin-dependent kinase pathway (CDKN1B, CDKN2A, CDKN2B, CDKN2C, CDK6), WNT signaling (CTNNB1, FAT1, GNAS, RNF43), mTOR signaling (MTOR, PIK3CA, PTEN, FLCN, TSC2), RTK or receptor tyrosine kinase signaling (BRAF, ERBB3, EPHA5, FGFR3, FRS2, FLT4, KRAS, NRAS, NTRK3).

**Figure 1:**
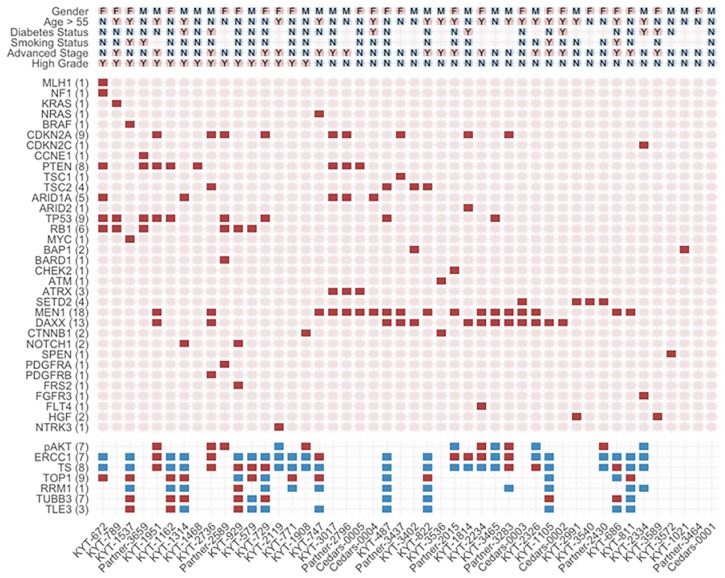
Heatmap illustrating subgroup associations across clinicopathologic variables using hierarchical clustering. Presence of select pathogenic genomic alterations are designated by solid red squares. Positive protein expression is designated by solid red squares while blue squares signify negative expression. Select clinicopathologic variables are designated by M for male gender, F for female gender, and Y or N for yes or no, respectively. Each column represents an individual case. Numbers of cases with genomic alterations or positive protein expression are displayed alongside row names.

Cluster analysis allowed generation of a heatmap to visualize putative correlations across clinicopathologic variables in our PanNET cohort (Figure 1). Notably, NGS alterations in *MEN1* and *DAXX*, both components of the chromatin remodeling pathway, tend to co-occur in non-metastatic, well-differentiated/intermediate grade PanNETS. NGS alterations in *TP53* and *RB1* tended to co-occur in high-grade tumors. *SETD2* was mutually exclusive of these alterations within other low-grade tumors. No other associations across clinicopathologic variables and genomic alterations/IHC expression reached statistical significance beyond those described in Table 3.

### Survival analysis

**Figure 2:**
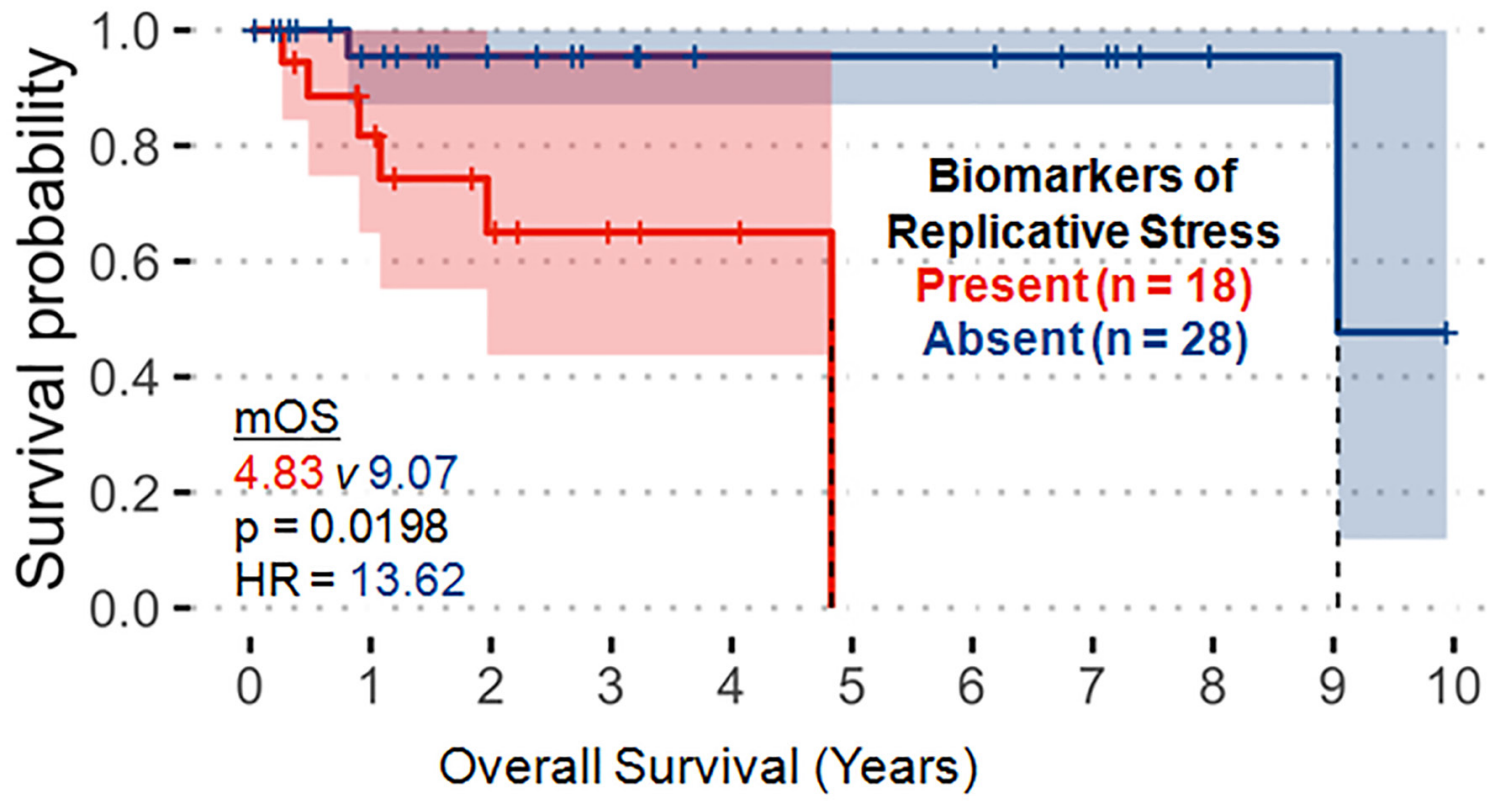
Molecular alterations associated with the replicative stress pathway (p53, RB1, ATRX, ATM, CCNE1, CHEK2, MYC) significantly correlate with decreased overall survival. These observations were primarily driven by TP53 and RB1 alterations.

Molecular alterations in the replicative stress pathway were the only variables demonstrating significant correlation with worse OS in our PanNET cohort (Figure 2, hazard ratio (HR) 13.62 [95% confidence interval (CI): 1.51-122.5], *p* = 0.0198). This finding was primarily driven by alterations in *TP53* and *RB1*. No variables were significantly correlated with progression-free survival (PFS) in our analysis.

## DISCUSSION

Beyond well-established germline mutations, PanNETs are increasingly recognized as molecularly-driven tumors with somatic mutations affecting chromatin remodeling (*MEN1*, *DAXX*, and *ATRX*) and mTOR signaling (*PTEN* and *TSC1/2*) as key oncogenic drivers [5]. Associations between clinicopathologic factors and genomic and proteomic signatures, however, remain poorly described and most large-scale studies have limited their focus on correlating *DAXX*/*ATRX* to prognostic variables [3, 4, 6, 7]. To the best of our knowledge, we are among the first to evaluate associations of molecular alterations across a spectrum of clinicopathologic variables and identify co-occurring pathogenic mutations and altered molecular pathways of potential clinical significance in PanNETs.

### Alterations in MEN1 and DAXX correlate with prognostic variables

Tumor stage and grade have long been known to be the most important independent predictors of survival in PanNETs [8, 9]. Our correlative analyses identified several significant associations with these 2 prognostic variables (Table 3). Alterations in *MEN1* (NGS) were less associated with having high-grade PanNETs and metastatic disease at diagnosis. NGS alterations in *MEN1* and *DAXX*, both components of the chromatin remodeling pathway, tend to co-occur in non-metastatic and well-differentiated/intermediate-grade PanNETs as visualized by hierarchical clustering (Figure 1), and a significant association between *MEN1* and *DAXX* was reached on pairwise comparisons (Table 3). Mutations in *ATRX* have been shown to be mutually exclusive to *DAXX* mutations and were excluded from our analysis [4].

Our potential positive prognostic signature with *MEN1* and *DAXX* alterations by NGS are consistent with an earlier NGS study that showed that alterations in *MEN1*, *DAXX*/*ATRX*, or the combination of were significantly associated with prolonged survival, in contrast to tumors lacking such mutations, in a metastatic PanNET cohort [3]. In predominantly non-metastatic and well-differentiated/intermediate grade PanNETs, however, loss of *DAXX*/*ATRX* by IHC correlated with higher tumor grade and stage, risk for metastasis, and worse survival [6, 7]. Recently, the ICGC analysis identified that median overall survival (OS) was not significantly different between *DAXX*/*ATRX*-mutant and -wild-type tumors, although *DAXX*/*ATRX*-mutant World Health Organization (WHO) grade 2 tumors had significantly worse OS compared to wild-type tumors [4]. Further study is needed to resolve this controversy regarding the prognostic value of chromatin remodeling alterations including *MEN1*, *DAXX*, and *ATRX*, which may be tumor stage and grade-specific. *SETD2* was mutually exclusive of *DAXX* and *MEN1* alterations within low-grade tumors in our cohort; the prognostic significance of this alteration may need further validation in larger PanNET cohorts.

### Replicative stress pathway alterations as primarily driven by TP53 and RB1 correlate with worse overall survival

On our OS analysis, only alterations in the replicative stress pathway were significantly correlated with worse survival (Figure 2). Having an altered replicative stress pathway correlated with high-grade disease as well. Notably, these observations were primarily driven by *TP53* (NGS) and *RB1* (NGS) alterations, of which both were significantly associated with high-grade disease on pairwise comparison (Table 3) and co-occurred in high-grade tumors (Figure 1). Abnormal expression of TP53 and RB1 have been shown to define poorly differentiated PanNETs with worse disease-specific survival compared to loss of DAXX or ATRX in well-differentiated PanNETs which have much better survival [10].

To sum, alterations in the DNA replicative stress pathway, in particular *TP53* and/or *RB1* alterations, significantly correlate with a negative prognostic signature. Targeting of DNA damage repair /replicative stress pathways with systemic therapies such as poly(ADP-ribose) polymerase (PARP) inhibitors or checkpoint kinase 1 (CHEK1/CHK1) inhibitors or cell cycle pathways with CDK inhibitors have yet to be as established in PanNETs as in other cancers, but our findings suggest a potentially targetable subset of PanNET patients with DNA replicative stress pathway alterations and poor prognosis that are primarily driven by *RB1* and *TP53* [11–13]. Future investigation is warranted to test these hypotheses in PanNETs.

### Other associations of potential clinical significance

We identified several other significant correlations of potential clinical importance on pairwise comparisons (Table 3): history of diabetes with CDK (positive correlation) or replicative stress pathway alterations (negative correlation), age >54 with non-metastatic disease, ERCC1 expression (IHC) with *DAXX* or *MEN1* alterations (NGS), and *PTEN* (NGS) with *ARID1A* or *TP53* alterations (NGS). ERCC1 expression has been shown to be putative prognostic and predictive biomarkers across various solid malignancies [14, 15]. In our series, ERCC1 expression positively correlated with favorable prognostic alterations (*DAXX* and *MEN1*). In separate studies of PanNET patients, loss of *PTEN* and *ARID1A* were associated with poorer prognosis [16, 17]. This appears consistent with our findings where *PTEN* co-occurred with *ARID1A* as well as the unfavorable prognostic *TP53* alteration.

The rationale behind the associations between a history of diabetes and alterations in the CDK or replicative stress pathway are unclear, but an altered replicative stress pathway portended to a poorer OS in our cohort (Figure 2). Age >54 was significantly less associated with metastatic disease at diagnosis (Table 3). This finding is contradictory to evidence where increasing age has historically portended a worse prognosis in large PanNET cohorts [8, 9]. Instead, this finding is more likely due to the fact that the majority of our metastatic or high-grade PanNET patients were age ≤54 (Figure 1). The tendency for these molecular alterations and clinicopathologic features of various prognostic/predictive significance to co-occur in our cohort are hypothesis generating and provide impetus for subsequent validation in larger cohorts to define combinations of genomic/proteomic signatures of greatest prognostic and/or predictive value in PanNETs.

Lastly, it should be noted that this study was limited by its retrospective design and heterogeneity across patient characteristics and tumor biology. Our decision to include pathogenic variations only for analysis likely further limited our sample size though we believed this decision was justified in conducting our survival and correlative analyses to prognostic variables. Nevertheless, we identified several molecular signatures through multi-omic profiling of potential clinical significance that are hypothesis generating and can inform future investigations of larger and ideally prospective design. Increasing efforts to molecularly profile PanNETs will enhance the possibility to stratify PanNET patients into molecularly-defined treatment subgroups based on prognostic and predictive biomarkers.

## MATERIALS AND METHODS

### Study population and tumor samples

**Figure 3:**
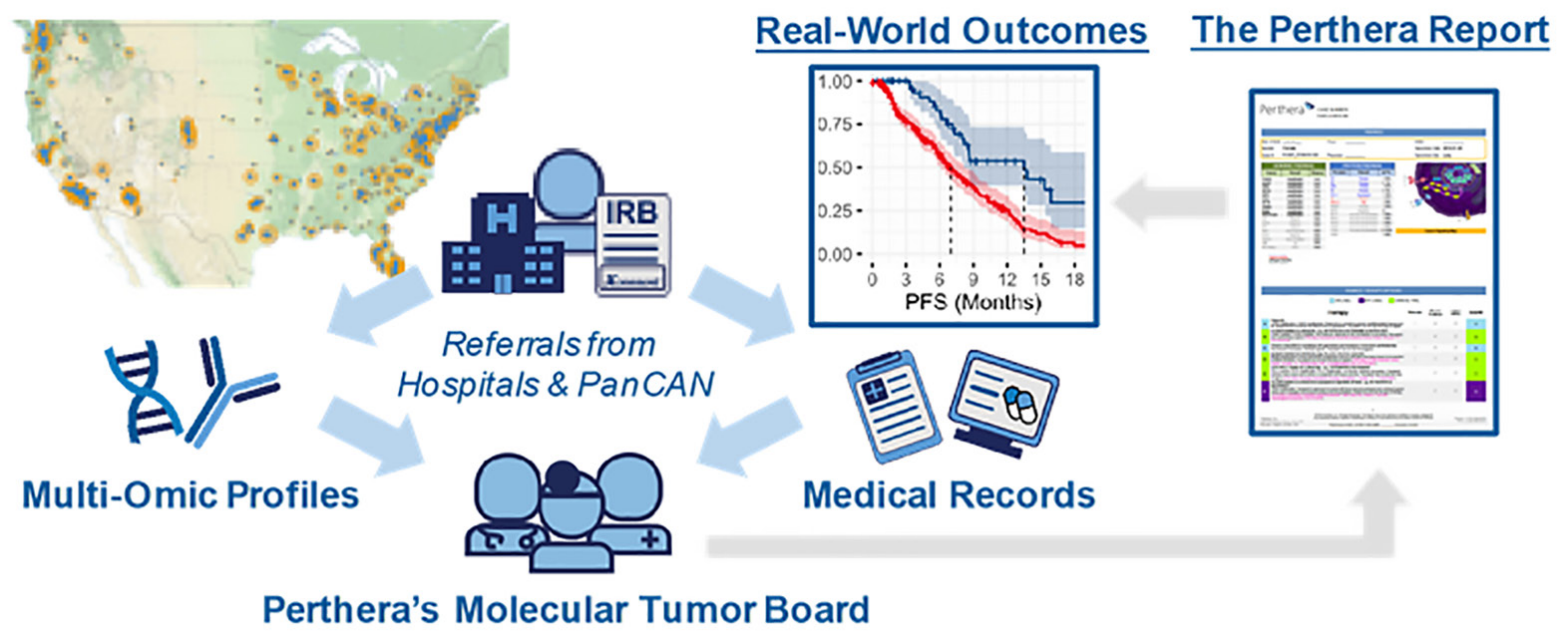
Perthera’s precision oncology process for curating real-world outcomes leverages the expertise of a molecular tumor board for actionability assessment. Perthera’s institutional review board-approved registry includes pancreatic neuroendocrine tumor patients from multiple cancer centers and patients referred via the Pancreatic Cancer Action Network’s Know Your Tumor^®^ (KYT) program. Real-world outcomes were collected after cases were reviewed by Perthera’s Molecular Tumor Board to deliver personalized treatment recommendations based on molecular findings. Next-generation DNA sequencing (NGS) was performed by Foundation Medicine and proteomics/immunohistochemistry (IHC) by Neogenomcs Inc. or Caris Life Sciences, Inc.

Informed consent was obtained from patients with pancreatic cancers through an IRB-approved protocol that facilitated the collection of real-world outcomes for research purposes. Patients diagnosed with histologically-confirmed pancreatic neuroendocrine tumors (PanNETs) were referred to Perthera, Inc. (McLean, VA) between 12/2014–12/2018 by their treating physician at a partnering institution or via the Pancreatic Cancer Action Network’s Know Your Tumor^®^ (KYT) program (Figure 3). Participating patients received personalized treatment recommendations from Perthera’s molecular tumor board based on CLIA/CAP-certified genomic and proteomic testing results that were ordered from commercial testing laboratories. The analysis cohort in this study included histologically confirmed cases of PanNET in which NGS testing results were considered adequately sensitive. Multiplatform profiling data was also available for all but 19 PanNET cases which included IHC protein testing results for chemopredictive markers (e.g. RRM1, ERCC1, TYMS) and other predictive markers (PD-L1, HER2). All PanNET cases were categorized based on the WHO criteria [18]. There were no exclusions to site of biopsy, previous treatment or lines of prior therapy, disease stage, medical comorbidities, or performance status.

### Multiplatform profiling

The processing of formalin-fixed paraffin-embedded (FFPE) tissues was performed in accordance to requirements per specific platform as previously described [19, 20]. NGS was performed on submitted tumor samples through FoundationOne (Foundation Medicine, Inc., Cambridge, MA). The FoundationOne NGS assay, which detects all 4 classes of genomic alterations including base substitutions, insertions and deletions, copy number alterations (CNAs), and rearrangements, has been previously described and validated [19]. Analysis by IHC was performed on FFPE tumor tissues through Caris Life Sciences (Phoenix, AZ) and NeoGenomics, Inc. (Fort Myers, FL) using commercially available detection kits and autostainers along with commercially available antibodies as previously described [20–22].

### Statistical analysis

Real-world outcomes data including demographics information, clinicopathologic factors, and survival metrics were abstracted from medical records and analyzed alongside molecular testing results from commercial laboratories. Descriptive statistics and other statistical analyses were computed in an R/Bioconductor programming environment. OS and PFS were evaluated for pairwise correlative analyses between individual pathogenic alterations or altered molecular pathways and clinicopathologic variables. Odds ratios (ORs) and 95% CIs were calculated using Cox proportional hazards regression as implemented in the R *survival* package. Statistical significance was defined by a Fisher’s exact test (two-tailed) *p* ≤ 0.05.

Pathogenic alterations were grouped into the following molecular pathways: replicative stress (p53, RB1, ATRX, ATM, CCNE1, CHEK2, MYC), chromatin remodeling (ARID1A, ARID2, ASXL1, CHD2, CTCF, DAXX, KMT2D, MEN1, MLL3, PBRM1, SETD2, TET2), cyclin-dependent kinase (CDKN1B, CDKN2A, CDKN2B, CDKN2C, CDK6), WNT signaling (CTNNB1, FAT1, GNAS, RNF43), mTOR signaling (MTOR, PIK3CA, PTEN, FLCN, TSC2), and receptor tyrosine kinase (RTK) signaling (BRAF, ERBB3, EPHA5, FGFR3, FRS2, FLT4, KRAS, NRAS, NTRK3). Only pathogenic alterations were included for correlative and survival analyses, and variants of uncertain significance (VUS) were excluded.
